# Health Literacy, Self-Perceived Health, and Substance Use Behavior among Young People with Alcohol and Substance Use Disorders

**DOI:** 10.3390/ijerph18084337

**Published:** 2021-04-19

**Authors:** Gabriela Rolova, Beata Gavurova, Benjamin Petruzelka

**Affiliations:** 1 Department of Addictology, First Faculty of Medicine and General University Hospital in Prague, Charles University, Apolinářská 4, 128 00 Prague 2, Czech Republic; gabriela.rolova@lf1.cuni.cz (G.R.); benjamin.petruzelka@lf1.cuni.cz (B.P.); 2 Center for Applied Economic Research, Faculty of Management and Economics, Tomas Bata University in Zlín, Mostní 5139, 760 01 Zlín, Czech Republic

**Keywords:** health literacy, HLS-EU-Q, alcohol use disorder, substance use disorders, residential addiction treatment, multiple regression

## Abstract

Licit and illicit substance use is one of the major public health issues with severe negative health consequences for individuals and society. Health literacy is essential for improving one’s health and navigation in the healthcare system. However, the evidence of health literacy in people with substance use disorders is limited. This study aims to examine health literacy and its socio-demographic, health-related, and substance use-related correlates in young people with alcohol (AUD) and substance use disorders (SUD). In this study, cross-sectional data of young people undergoing addiction treatment for AUD (*N* = 201, mean age 37.6) and SUD (*N* = 165, mean age 31.1) were used. Health literacy was assessed using the HLS-EU-Q47. Simple and multiple linear regression was performed to estimate the correlates of health literacy. In total, 37.8% of participants with AUD and 41.8% of SUD had limited health literacy. In participants with AUD, living condition factors, self-perceived health indicators, and frequency of alcohol use showed a significant effect on health literacy. In participants with SUD, financial factors, self-perceived health indicators, and injection sharing showed a significant effect. Increasing health literacy might contribute to improved health outcomes and decreased high-risk substance use-related behavior in people undergoing addiction treatment.

## 1. Introduction

Licit and illicit substance use is one of the main public health issues with severe negative health consequences for individuals and society [1,2]. Harmful substance use is causally linked to hundreds of physical and mental illnesses and is, therefore, classified among the greatest risk factors for preventable morbidity and mortality [3,4]. Morbidity and mortality related to substance use also affect economic parameters, e.g., in the form of lost productivity and higher cost of diagnostic and treatment processes [5,6,7]; total expenditure attributable to alcohol, tobacco, illicit drugs, and gambling is estimated at up to 3.05–3.15% of GDP in the Czech Republic [8]. However, harmful substance use not only has negative health consequences but also affects social and family relationships, whose quantification for individuals and society is methodologically highly complex [9].

Substance use is a complex phenomenon that has been subjected to extensive research for decades. Its intensity is determined by the complexity and variability of individual socio-economic factors, lifestyle dynamics, globalization processes, and other factors; thus, high heterogeneity in the focus of these studies situated in medical, social, economic, psychological, and other disciplines is observable [10,11,12]. Due to severe negative consequences of the persisting trend of harmful substance use in the population, it is necessary to verify existing knowledge about this phenomenon and seek new ones. The current trend is to investigate complex risk factors that can create strong negative links and complicate prevention or even treatment of substance use disorders.

Health literacy, a multidimensional concept addressing the use of health information, has recently been associated with various risky health behaviors, including substance use [13,14,15,16,17,18]. Currently, several conceptual frameworks of health literacy exist, as the concept has evolved. Health literacy was originally linked to the basic literacy skills of reading, writing, and numeracy in a medical context [19,20]. Recently, a shift to a broader—comprehensive—concept, including a wide range of individual, social, and cognitive competencies, is noticeable [16].

Previous evidence showed that health literacy decreases with age and lower levels of education. Higher percentages of low health literacy were found between socioeconomically disadvantaged people and those who belong to ethnic minorities [21,22]. Moreover, health literacy is considered an important determinant of individual and population health [19,23,24]. Low health literacy is consistently related to poor health status, higher mortality rates, more hospitalization and emergency care use, lower use of preventive activities, poor medication adherence, and poor ability to interpret written health information [23]. Therefore, promoting health literacy can potentially prevent risky health behavior, improve health outcomes, decrease health inequalities, and improve navigation in the healthcare system.

People with alcohol (AUD) or substance use disorder (SUD) are at risk of lower health literacy due to multiple negative health, psychosocial, and economic factors related to substance use behavior [3,25]. Degan et al. (2019) examined health literacy and its association with a number of socio-economic and health-related factors in a mixed sample of people with AUD and SUD (*N* = 298), finding the prevalence of inadequate health literacy 87%. Low health literacy was associated with higher psychological distress, poor social support, mental health, and quality of life [26]. Rolová et al. (2018) assessed health literacy in people with AUD undergoing inpatient and outpatient addiction treatment (*N* = 113), finding the prevalence of low health literacy at 46.9% [27]. Most recently, Dahlman et al. (2020) examined health literacy in patients in opioid substitution treatment (OST) (*N* = 286, including the invalid questionnaires). They reported a prevalence of low health literacy of 22%, but the actual prevalence is likely higher because they added one third of invalid questionnaires into the calculation [28]. Neither did Rolová et al. (2018) nor Dahlman et al. (2020) find any association between health literacy and investigated socio-economic factors [27,28].

Overall, to our knowledge, only three studies examined multidimensional health literacy and its correlates in a disadvantaged population of people with substance use disorders. Previous evidence is limited by bivariate analysis of the data, without controlling for the contribution of other variables in the significant relationship.

Therefore, we aim to (1) describe health literacy in young people with AUD and SUD using a multidimensional measuring tool and (2) investigate the association between health literacy and socio-demographic characteristics, self-perceived health indicators, and substance use behavior in both samples.

## 2. Methods

In this cross-sectional study, we examine and compare the health literacy in young people undergoing residential addiction treatment for AUD and SUD in the Czech Republic. We used part of the data (*N* = 394) from a cross-sectional survey on health literacy in people undergoing residential addiction treatment. For detailed methodology, see Rolová (2020) [29].

### 2.1. Study Sample and Data Collection

Sampling and data collection were conducted between May and December 2019. Institutions of residential addiction treatment (*N* = 50), i.e., detoxification units with dedicated detoxification beds offering medical detoxification programs, state-run psychiatric hospitals offering long-term institutional treatment, and therapeutic communities offering socio-therapeutic care for individuals with addiction, served as a sampling frame. Of those, 16 (32%) gave us permission to carry out the recruitment of the participants.

Original inclusion criteria included men or women, 15 years old and older, fluent in Czech, and diagnosed with alcohol and other substance use disorders or addictive behaviors. In the present study, only those aged between 18 and 45 years and with a diagnosis of AUD or SUD were included in the analysis.

Data were collected on-site of the involved facilities through anonymous paper-and-pencil questionnaires from all eligible individuals. Participants gave oral consent concerning their involvement in the questionnaire survey prior to the data collection and further expressed their willingness to participate in the survey by completing and submitting the questionnaires to the administrator. Written informed consent requiring personal data of participants was not collected to preserve the anonymity of those involved.

Prior to the statistical analysis, participants were divided into one of two study groups, according to the following criteria: The AUD sample comprises those who reported alcohol as their drug of the first; the SUD sample comprises those who reported any of the illicit substances (cannabinoids, MDMA/ecstasy, methamphetamine, and other amphetamines, cocaine, heroin, buprenorphine and methadone, hallucinogens, inhalants, prescription medications, new psychoactive substances, other) as their drug of the first choice.

### 2.2. Dependent Variable

This study follows the conceptual framework elaborated by Sørensen [16]. The health literacy of the participants was assessed using the 47-item version of the European Health Literacy Survey Questionnaire (HLS-EU-47) [30]. A Czech translation of the HLS-EU-Q47 was officially acquired from the National Institute of Public Health of the Czech Republic (Ref. PID UK1LF18G/03010 001).

The questionnaire assesses the perceived difficulty of various health-related tasks on a 4-point Likert scale ranging from “very easy” to “very difficult”. Health literacy score (general health literacy index) and three additional indices for sub-domains of health literacy—healthcare, disease prevention, and health promotion—were calculated using the following formula:

*Index* = (*mean* − 1) × (50/3)

where *Index* is the specific index calculated, *mean* is the mean of all participating items for each individual, 1 is the minimal possible value of the mean, 3 is the range of the mean, and 50 is the chosen maximum value of the new metric. Index 0 represents the lowest possible health literacy and 50 the highest health literacy [31].

In addition, four levels of health literacy were defined according to the recommended cut-offs as “inadequate” (0–25), “problematic” (>25–33), “sufficient” (>33–42), and “excellent” (>42–50) to describe the distribution of health literacy in the study samples. The inadequate and problematic levels correspond to “limited health literacy”; the sufficient and problematic levels correspond to “adequate health literacy” [31].

### 2.3. Independent Variables

Measurement of socio-demographic characteristics, self-perceived health indicators, and substance use behavior of the participants are described in Rolová (2020) [29]. Socio-demographic characteristics include gender, age, marital status, housing condition, household size, educational attainment, employment status, household net income, debt situation, and size of place of residence.

Self-perceived health indicators of general health status, mental health status, physical condition, and quality of life were measured by single-item questions with the five Likert-type responses (1—bad, 2—rather bad, 3—neither bad nor good, 4—rather good, 5—good). Self-perceived health indicators were treated as continuous variables in regression analysis. Psychiatric comorbidity was assessed by self-report.

Substance use behavior includes cigarette smoking, past-year frequency of alcohol use, binge drinking (use of 5 or more glasses of alcohol on one occasion), and alcohol intoxication, lifetime and past-year illicit drug use, the drug of the first choice, age at onset of alcohol use, alcohol intoxication, marijuana use, and illicit drug use, and a number of premature addiction treatment terminations. Participants with substance use disorders were asked to report the preferred method of drug administration, age at onset of intravenous application, injection sharing, and drug-related infectious diseases.

### 2.4. Statistical Analysis

We analyzed the data using descriptive statistics, correlation analyses, and linear regression. Indices of health literacy were calculated and categorized to describe the distribution of health literacy in the study samples. Pearson’s chi-square test (for categorical variables) and one-way ANOVA (for continuous variables) were used to determine the differences between the study samples.

Linear regression was performed to estimate health literacy correlates. Simple (univariate) linear regression was used to investigate the relationship between the score of the health literacy assessment (dependent variable) and socio-demographic characteristics, self-perceived health indicators, and substance use behavior. Multiple regression analysis was used to explain the contribution of variables in the health literacy score when controlled for other variables. We did not adjust the multiple analysis for all significant variables to prevent over-fitting of the regression model. The variables entering the regression model were selected based on a priori theoretical knowledge, the empirical importance of variables for this research, and with the aim that each variable category is represented by at least one variable. Previous studies in general and clinical populations found a relationship between health literacy and gender, age, employment status, financial deprivation, and mental health [21,22,26,32]. Regarding the substance use behavior, we included frequency of alcohol use (for AUD) and injection sharing (for SUD) as our variables of interest.

The adjusted R-squared was used to measure the proportion of variation in health literacy score explained by correlates. In all levels, the variables with the alpha level of 0.50 were considered to be statistically significant outcomes. Statistical analyses were performed using IBM SPSS Statistics 23 (IBM Corp., Armonk, NY, USA).

## 3. Results

Overall, 394 young people (18–45 years old) undergoing residential addiction treatment, of which 201 were people with AUD (24.9% of women, mean age 37.6, median 39) and 165 people with SUD (19.4% of women, mean age 31.1, median 31), were selected for this study. The majority in both samples were men, non-married, with stable housing, living in multi-person households, and with household net income between EUR 1317–2249. In terms of socio-demographic characteristics, study samples differed in terms of age, educational attainment, marital and employment status, and debt situation (Table 1).

### 3.1. Distribution of Health Literacy

Participants with AUD achieved a mean score (i.e., general health literacy index) of 34.8 (SD = 6.4) out of a potential 50 in HLS-EU-Q47. Overall, 6% of participants with AUD had inadequate, 31.8% problematic, 47.8% sufficient, and 14.4% excellent health literacy. When the scale was dichotomized into two levels, 37.8% of participants with AUD fell into the category of limited health literacy. Participants achieved a mean score of 37.7 (SD = 6.5) in healthcare, 34.6 (SD = 7.5) in disease prevention, and 32.1 (SD = 7.9) in health promotion.

Participants with SUD achieved a mean score of 34.5 (SD = 6.9) in HLS-EU-Q47. Overall, 9.1% of participants with SUD had inadequate, 32.7% problematic, 41.8% sufficient, and 16.4% excellent health literacy; 41.8% of participants fell into the category of limited health literacy. Participants achieved a mean score of 36.6 (SD = 6.5) in healthcare, 34.4 (SD = 8.2) in disease prevention, and 32.5 (SD = 8.6) in health promotion.

There were no statistically significant differences in health literacy scores between the samples (see Table 1).

### 3.2. Correlates of Health Literacy in People with AUD

Simple linear regression (Table 2) showed a negative significant relationship between health literacy and household condition (*p* = 0.008), household size (*p* = 0.024), employment status (*p* = 0.028), alcohol use (*p* = 0.032), and binge drinking (*p* = 0.034). A positive relationship was found between health literacy and general health status (*p* = 0.009), mental health status (*p* = 0.001), physical condition (*p* = 0.002), and quality of life (*p* = 0.002). Participants with stable housing, living in a multi-person household, employed or short-term unemployed, drinking less than daily, binge drinking less than daily, with better general health status, mental health status, physical condition, and quality of life scored significantly higher in HLS-EU-Q47.

Multiple linear regression (see Table 2) showed that after adjusting for gender, age, housing condition, employment status, mental health status, and alcohol use, health literacy remained significantly associated with housing condition (b = 4.06, 95% CI [−7.47, −0.66], *p* = 0.020), alcohol use (b = 2.02, 95% CI [−3.85, −0.19], *p* = 0.031), and mental health status (b = 1.40, 95% CI [0.56, 2.25], *p* = 0.001). Employment status dropped out of significance.

In people with AUD, multiple regression explained 10.4% of the variance in health literacy score (R^2^adj = 0.104).

### 3.3. Correlates of Health Literacy in People with SUD

Simple linear regression (Table 3) showed a positive significant relationship between health literacy and household net income (*p* = 0.040), general health status (*p* = 0.028), mental health status (*p* = 0.001), physical condition (*p* = 0.023), and quality of life (*p* = 0.033). A negative significant relationship was found between health literacy and debt situation (*p* = 0.021) and injection sharing (*p* = 0.011). Participants with higher household net income, better general health status, mental health status, physical condition, quality of life, without debts, and not involved in injection sharing scored significantly higher in HLS-EU-Q47.

Multiple linear regression (see Table 3) showed that after adjusting for gender, age, debt situation, mental health status, and injection sharing, health literacy remained significantly and positively associated only with mental health status (b = 1.58, 95% CI [0.58, 2.57], *p* = 0.002). Debt situation and injection sharing dropped out of significance.

In people with SUD, multiple regression explained 11.0% of the variance in health literacy score (R^2^adj = 0.110).

## 4. Discussion

Our study focused on health literacy and its correlates in young people undergoing addiction treatment for AUD and SUD. Using the HLS-EU-Q47, we comprehensively assessed the health literacy of a well-defined clinical population of young people with substance use disorders in Central Europe. We used multiple regression to examine a wide range of health literacy correlates, focusing specifically on health- and substance use-related factors.

In total, 37.8% of participants with AUD and 41.8% with SUD had limited health literacy when assessed with the HLS-EU-Q47. Previous studies in substance-using populations using a multidimensional approach to health literacy are inconsistent on this matter; the reported prevalence of lower health literacy ranges between 22 and 87% [26,27,28]. This inconsistency may be related to the different methodologies used or the characteristics of study samples in these studies.

We did not observe any significant differences in health literacy scores of participants with AUD and SUD despite their differences in socio-demographic backgrounds. Individuals with AUD and SUD in long-term addiction treatment (treatment duration is usually between 3–12 months) undergo the treatment process together. They are in daily contact with healthcare providers and regularly educated on various health topics. It is, therefore, reasonable to assume that receiving addiction treatment may improve patients’ health literacy to the point where differences in their health-related competencies are eliminated. It would be interesting to explore to what extent different addiction treatment programs can promote the health literacy of the patients.

In participants with AUD, lower health literacy was associated with being homeless, living alone, and being long-term unemployed. In accordance with our findings, previous research found that homeless persons with mental illness tend to have low health literacy [32]. Homeless persons are disadvantaged in access to healthcare and lack the medical support of healthcare professionals [33,34], which are the factors known to negatively affect health literacy [35]. In terms of the relationship between health literacy and household size, this finding highlights the importance of social and family relationships in the transfer of health-related information and skills [36]. As regards the relationship between health literacy and employment status, long-term unemployment is consistently linked to poor health status, mental illness in particular [37], which is associated with low health literacy [23,26]. In addition, lower health literacy was associated with daily drinking and daily binge drinking in people with AUD, indicating the higher severity of AUD in individuals with low health literacy. We assume that the negative effects of excessive alcohol consumption on cognitive functioning may be the reason for the lower health literacy of those with severe AUD [38]. However, this must be confirmed by other studies that will examine this relationship using the standardized multi-item instruments to measure the severity of AUD.

In participants with SUD, lower health literacy was associated with lower household net income, being burden with debts, and using injection materials previously used by other users. In a previous large-scale population-based study, financial deprivation was found to be one of the strongest predictors of low health literacy [22]. It could be explained by the fact that socioeconomically disadvantaged individuals may not have the financial resources to make healthier choices, e.g., to buy healthy food, health-related literature, attend sport or educational courses, etc., [39,40]. In terms of health literacy and injection sharing, the relationship indicates that substance users with low health literacy might be more inclined to a certain high-risk substance use behavior. Increasing health literacy and drug-related literacy in people with SUD might result in less risky substance use and reduce the spread of drug-related infectious diseases. However, the association between health literacy and the burden of drug-related infectious diseases was not confirmed in this study, more evidence is, therefore, needed on this relationship.

In accordance with other studies in diverse and addicted populations, self-perceived general health status, mental health status, physical condition, and quality of life were positively associated with health literacy in both samples of participants with AUD and SUD [13,23,26]. In both groups, mental health status was found to be the strongest predictor of health literacy. The relationship between health literacy and health outcomes is well established; health literacy is recognized as an independent social determinant of health. Moreover, the evidence suggests that the relationship between health literacy and health indicators is at least partially mediated by health knowledge, self-efficacy, norms, and perceived stigma [23].

Our findings support both the improvement of existing health literacy-promoting programs as well as the development of new ones tailored to the needs of the patients in the healthcare setting. Health literacy-promoting programs, as a quality, comprehensive, long-term tool for improving the health of the population and increasing the efficiency of the healthcare service, must be developed conceptually and systemically. It is to be directly linked to existing concepts of health literacy and tailored to the demographic, health, geographical, social, and other characteristics of its recipients. There is potential in well-designed health literacy-promoting programs to eliminate the emergence of repeated morbidity that impact the healthcare system’s economic resources, which are exhaustible.

Programs of addiction treatment provide therapeutic care with varying durations; therefore, it is assumed that longer use of therapeutic healthcare processes will also affect patients’ health literacy. In treatment programs of shorter duration, there is also a space to promote the health literacy of patients, but it is important to identify the factors that most influence it.

Investigation of patient’s socio-economic characteristics that might influence health literacy during the treatment regardless of its length or type of health or social service program also comes to attention. Unstable and absent family background or housing, long-term unemployment, and income loss remain strong risk factors for returning to addictive behavior, prompting the need to examine new social trajectories in relation to health literacy among patients during treatment. Therefore, we suggest that future research should continue to investigate the correlates of health literacy in people with addictions with the special emphasis on health indicators, living conditions, and financial factors.

Finally, this study has several limitations that must be acknowledged. The cross-sectional design of the study does not allow causality between health literacy and its correlates to be established. Regarding the measurement tool, health literacy was measured using the self-administered tool. Subjective questionnaires are known to be prone to social desirability and recall bias [41]; therefore, the level of health literacy in the participants may not be estimated correctly. Moreover, Finbråten (2018) recently pointed out some psychometric shortcomings (violation of multidimensionality and response dependence) of HLS-EU-Q47 [42]. On the other hand, other previous studies tested psychometric properties of HLS-EU-Q47 with satisfactory outcomes [30,43]. The Czech translation of HLS-EU-Q47 was not systematically validated for the Czech population. However, the Czech version of the questionnaire was tested in the representative population-based study of Kučera et al. (2016) [44]. As for the study sample, both study samples are rather small; therefore, studies with larger samples are needed to confirm our results. Nevertheless, given the specific characteristics of this population, the study may offer valuable insight into this issue. Self-selection of study participants could have resulted in biased results; although, the overall response rate to the recruitment process was high. The proportion of those involved from all eligible individuals was 86%.

## 5. Conclusions

In this study, we examine health literacy in a clinical population of young people with substance use disorders in the Czech Republic. Our results suggest that a considerable proportion of young people undergoing addiction treatment with AUD and SUD might not be able to use health information to take care of their health and navigate the healthcare system effectively. Health literacy should be systematically promoted in residential addiction treatment programs to improve the health outcomes of patients. We identified a number of related factors that might influence or be influenced by health literacy in people with substance use disorders. Furthermore, this study highlights the importance of the investigation of complex risk factors in the research of substance use.

## Figures and Tables

**Table 1:** Socio-demographic, health-related, and substance use-related characteristics of participants with AUD and SUD and the differences between the study samples.

	AUD (*N* = 201)		SUD (*N* = 165)		
Characteristics	*N* (* mean)	% (* *SD*)	*N* (* mean)	% (* *SD*)	*p*
Health literacy					
General health literacy *	34.8	6.4	34.5	6.9	0.674
Healthcare *	37.7	6.5	36.6	6.5	0.110
Disease prevention *	34.6	7.5	34.4	8.2	0.794
Health promotion *	32.1	7.9	32.5	8.6	0.615
Gender					0.260
Man	151	75.1	133	80.6	
Woman	50	24.9	32	19.4	
Age *	37.6	5.9	31.1	6.4	<0.001
Type of treatment					<0.001
Detoxification	33	16.4	25	15.2	
Long-term inpatient care	136	67.7	98	59.4	
Therapeutic community	11	5.5	35	21.2	
Follow-up inpatient care	21	10.4	7	4.2	
Marital status					<0.001
Married	36	17.9	6	3.6	
Other	165	82.1	159	96.4	
Housing condition					0.009
Stable housing	182	90.5	131	79.4	
Without home	16	8.0	28	17.0	
Household size *	2.26	1.3	2.63	1.5	0.178
Single-person	79	39.3	49	29.7	
Multi-person	113	56.2	106	64.2	
Educational attainment					<0.001
Primary	29	14.4	68	41.2	
Secondary and higher	171	85	96	58.2	
Employment status					<0.001
Employed	125	62.2	69	41.8	
<6 months	46	22.9	35	21.2	
≥6 months	22	10.9	46	27.9	
Household net income					0.159
EUR <562	34	16.9	34	20.6	
EUR 563–1316	81	40.3	50	30.3	
EUR 1317–2249	54	26.9	42	25.5	
EUR >2249	20	10.0	25	15.2	
Debt situation					<0.001
Debts	65	32.3	87	52.7	
No debts	135	67.2	77	46.7	
Psychiatric comorbidity					0.136
Yes	41	20.4	45	27.3	
No	157	78.1	117	70.9	
General health status *	3.7	1.1	3.8	1.0	0.137
Bad or rather bad	25	12.4	19	11.5	
Neither bad nor good	60	29.9	42	25.5	
Good or rather good	115	57.2	102	61.8	
Mental health status *	3.5	1.1	3.6	1.0	0.144
Bad or rather bad	35	17.4	26	15.8	
Neither bad nor good	64	31.8	43	26.1	
Good or rather good	101	50.2	94	57.0	
Physical condition *	3.6	1.0	3.7	1.0	0.124
Bad or rather bad	33	16.4	23	13.9	
Neither bad nor good	57	28.4	40	24.2	
Good or rather good	111	55.2	101	61.2	
Quality of life *	3.1	1.1	3.1	1.0	0.279
Bad or rather bad	59	29.4	41	24.8	
Neither bad nor good	72	35.8	66	40.0	
Good or rather good	69	34.3	57	34.5	
Cigarette smoking					0.512
Non-smoker	46	22.9	31	18.8	
<15 cigarettes	38	18.9	36	21.8	
≥15 cigarettes	108	53.7	95	57.6	
Alcohol use					<0.001
Less than daily	82	40.8	117	70.9	
Daily	114	56.7	46	27.9	
Binge drinking					<0.001
Less than daily	110	54.7	128	77.6	
Daily	86	42.8	33	20.0	
Alcohol intoxication					0.041
Less than daily	173	86.1	153	92.7	
Daily	23	11.4	9	5.5	
Lifetime illicit drug use					N/A
No	47	23.4	0	0	
Yes	92	45.8	152	92.1	
Past year illicit drug use					<0.001
No	103	51.2	6	3.6	
Yes	92	45.8	152	92.1	
First alcohol use *	15.2	3.6	13.6	2.5	0.024
First alcohol intoxication *	16.4	4.1	14.2	2.3	<0.001
First marijuana use *	18.0	5.0	14.8	2.4	<0.001
First illicit drug use *	20.0	5.2	17.7	3.8	<0.001
Premature treatment termination *	0.7	1.6	1.3	2.4	0.106
Intravenous drug administration					N/A
Yes	0	0	54	32.7	
No	201	100	109	66.1	
First intravenous administration *			19.1	4.1	N/A
Injection sharing					N/A
Yes			64	38.8	
No			97	58.8	
Drug-related infectious diseases					N/A
Yes			35	21.2	
No			126	76.4	

Note: AUD = alcohol use disorder; SUD = substance use disorder; N = number of cases; *SD* = standard deviation; *p* = *p*-value, * = continuous variable. Pearson’s chi-square test (for categorical variables) and one-way ANOVA (for continuous variables) were used to determine the statistical differences between the samples.

**Table 2:** Simple (univariate) and multiple linear regression models for health literacy (dependent variable) and socio-demographic, health-related, and substance use-related correlates for the sample of people with AUD.

	Univariate			Multiple (*N* = 186)		
Factor	b (95% CI)	SE	*p*	b (95% CI)	SE	*p*
Gender						
Woman	−0.01 (−2.06, 2.05)	1.0	0.994	−0.78 (−2.89, 1.33)	1.1	0.468
Man (ref.)						
Age	−0.03 (−0.18, 0.12)	0.1	0.708	−0.02 (−0.17, 0.13)	0.1	0.759
Type of treatment						
Detoxification	1.45 (−0.99, 3.90)	1.2	0.242			
Therapeutic community	−0.63 (−4.57, 3.32)	2.0	0.755			
Follow-up inpatient care	1.45 (−1.51, 4.40)	1.5	0.335			
Long-term inpatient care (ref.)						
Marital status						
Married	1.13 (−1.19, 3.44)	1.2	0.338			
Other (ref.)						
Housing condition						
Without home	−4.34 (−7.55, −1.12)	1.6	0.008	−4.06 (−7.47, −0.66)	1.7	0.020
Stable housing (ref.)						
Household size						
Single-person	−2.11 (−3.94, −0.29)	0.9	0.024			
Multi-person (ref.)						
Educational attainment						
Primary	−0.03 (−2.55, 2.49)	1.3	0.983			
Secondary and higher (ref.)						
Employment status						
Long-term unemployed	−3.18 (−6.00, −0.35)	1.4	0.028	−1.99 (−4.86, −0.88)	1.5	0.172
Employed/short-term unemployed (ref).						
Household net income	0.20 (−0.06, 0.46)	0.1	0.135			
Debt situation						
Debts	1.28 (−0.62, 3.17)	1.0	0.185			
No debts (ref.)						
Psychiatric comorbidity						
Yes	−0.33 (−2.54, 1.88)	1.1	0.770			
No (ref.)						
General health status	1.12 (0.29, 1.96)	0.4	0.009			
Mental health status	1.37 (0.55, 2.20)	0.4	0.001	1.40 (0.56, 2.25)	0.4	0.001
Physical condition	1.31 (0.47, 2.15)	0.4	0.002			
Quality of life	1.29 (0.47, 2.06)	0.4	0.002			
Cigarette smoking						
Non-smoker	−0.18 (−2.32, 1.96)	1.1	0.868			
<15 cigarettes	1.98 (−0.32, 4.27)	1.2	0.091			
≥15 cigarettes (ref.)						
Alcohol use						
Daily	−1.95 (−3.73, −0.17)	0.9	0.032	−2.02 (−3.85, −0.19)	0.9	0.031
Less than daily (ref.)						
Binge drinking						
Daily	−1.91 (−3.68, −0.15)	0.9	0.034			
Less than daily (ref.)						
Lifetime illicit drug use						
No	−0.62 (−2.73, 1.50)	1.1	0.566			
Yes (ref.)						
Past year illicit drug use						
Yes	0.57 (−1.24, 2.37)	0.9	0.538			
No (ref.)						
First alcohol use	0.07 (−0.19, 0.33)	0.1	0.594			
First alcohol intoxication	−0.05 (−0.28, 0.18)	0.1	0.685			
Premature treatment termination	−0.01 (−0.61, 0.59)	0.3	0.971			

Note: b: unstandardized coefficient; CI: confidence interval; SE: standard error; *p*: *p*-value; ref.: reference group.

**Table 3:** Simple (univariate) and multiple linear regression models for health literacy (dependent variable) and socio-demographic, health-related, and substance use-related correlates for the sample of people with SUD.

	Univariate			Multiple (*N* = 160)		
Factor	b (95% CI)	SE	*p*	b (95% CI)	SE	*p*
Gender						
Woman	−2.19 (−4.85, 0.48)	1.4	0.107	−2.29 (−4.96, 0.38)	1.4	0.093
Man (ref.)						
Age	0.05 (−0.11, 0.22)	0.1	0.529	0.06 (−0.11, 0.22)	0.1	0.484
Type of treatment						
Detoxification	1.32 (−1.73, 4.37)	1.6	0.395			
Therapeutic community	−1.15 (−3.84, 1.53)	1.4	0.397			
Follow-up inpatient care	1.02 (−4.31, 6.34)	2.7	0.707			
Long-term inpatient care (ref.)						
Housing condition						
Without home	−1.97 (−4.81, 0.87)	1.4	0.172			
Stable housing (ref.)						
Household size						
Single-person	−1.28 (−3.62, 1.05)	1.2	0.280			
Multi-person (ref.)						
Educational attainment						
Primary	−0.89 (−3.05, 1.28)	1.1	0.420			
Secondary and higher (ref.)						
Employment status						
Long-term unemployed	−0.75 (−3.16, 1.66)	1.2	0.541			
Employed/short-term unemployed (ref).						
Household net income	0.30 (0.01, 0.59)	0.1	0.040			
Debt situation						
Debts	−2.49 (−4.59, −0.29)	1.1	0.021	−2.01 (−4.15, 0.13)	1.1	0.066
No debts (ref.)						
Psychiatric comorbidity						
Yes	−2.19 (−4.55, 0.18)	1.2	0.070			
No (ref.)						
General health status	1.17 (0.13, 2.22)	0.5	0.028			
Mental health status	1.67 (0.68, 2.67)	0.5	0.001	1.58 (0.58, 2.57)	0.5	0.002
Physical condition	1.18 (0.16, 2.18)	0.5	0.023			
Quality of life	1.16(0.10, 2.22)	0.5	0.033			
Cigarette smoking						
Non-smoker	1.26 (−1.53, 4.05)	1.4	0.374			
<15 cigarettes	1.68 (−0.97, 4.32)	1.3	0.212			
≥15 cigarettes (ref.)						
Alcohol use						
Daily	0.37 (−2.01, 2.74)	1.2	0.760			
Less than daily (ref.)						
Binge drinking						
Daily	−0.91 (−3.54, 1,73)	1.3	0.498			
Less than daily (ref.)						
First alcohol use	0.20 (−0.25, 0.64)	0.2	0.391			
First alcohol intoxication	0.27 (−0.21, 0.75)	0.2	0.267			
First marijuana use	0.26 (−0.18, 0.70)	0.2	0.247			
First illicit drug use	0.16 (−0.12, 0.44)	0.1	0.258			
Premature treatment termination	0.06 (−0.41, 0.52)	0.2	0.813			
Intravenous drug administration						
Yes	−0.88 (−3.15, 1.39)	1.2	0.444			
No (ref.)						
First intravenous administration	−0,36 (−0.73, 0.02)	0.2	0.065			
Injection sharing						
Yes	−2.84 (−5.00, −0.67)	1.1	0.011	−1.89 (−4.06, 0.27)	1.1	0.086
No (ref.)						
Drug-related infectious diseases						
Yes	−0.16 (−2.79, 2.46)	1.3	0.902			
No (ref.)						

Note: b: unstandardized coefficient; CI: confidence interval; SE: standard error; *p*: *p*-value; ref.: reference group.

## Data Availability

The data presented in this study are available in aggregated form on request from the first author due to privacy reasons.
